# Nurses’ perceptions of critically ill patients’ eye care: a qualitative study in Iran

**DOI:** 10.1186/s12912-023-01176-1

**Published:** 2023-03-01

**Authors:** Maryam Askaryzadeh Mahani, Jamileh Farokhzadian, Fatemeh Bahramnezhad, Monirsadat Nematollahi

**Affiliations:** 1 Medical Mycology and Bacteriology Research Center, Kerman University of Medical Sciences, Kerman, Iran; 2 Nursing Research Center, Kerman University of Medical Sciences, Kerman, Iran; 3 Department of Critical Care Nursing, School of Nursing and Midwifery, Tehran University of Medical Sciences, Tehran, Iran; 4 Department of Pediatric and Neonatal Intensive Care Nursing, School of Nursing and Midwifery, Kerman University of Medical Sciences, Haft Bagh Alavi highway, Kerman, Iran

**Keywords:** Eye care, Intensive care unit, Content analysis, Nurse, Iran

## Abstract

**Background:**

Despite the high prevalence of ocular complications in patients admitted to the intensive care unit (ICU), eye care, as an important component of the care plan, has not received much attention from nurses. To improve the quality of eye care, the challenges and concerns of ICU nurses should be studied deeply. Thus, the present study aimed at exploring the challenges faced by ICU nurses in taking care of patients admitted to the ICU.

**Methods:**

The present conventional qualitative content analysis study was carried out on 11 nurses and 3 head nurses selected purposefully by observing the maximum variation. The data were collected through face-to-face, in-depth, and semi-structured interviews. All data were recorded, transcribed, and analyzed using the conventional content analysis method proposed by Graneheim and Lundman (Nurse Educ Today 24:105–12, 2004). The Max Q Data software 2020 was run to record the interviews and extract codes from the transcriptions.

**Results:**

The participants’ mean age was 37.14 ± 6.41 years and their average work experience in ICU was 10.29 ± 7.63 years. The core category that emerged from data analysis was “the lack of an evidence-based approach”, which was subdivided into five categories: “education, the missing link”, “nurses’ inadequate professional competence”, “unsafe nursing care”, “organizational requirements”, and “difficulty in eye care evaluation”.

**Conclusion:**

Evidence-based practice plays a minor role in the field of eye care in Iran, despite its critical importance. Thus, the Ministry of Health and Medical Education of Iran is recommended to adapt the clinical guidelines so that more attention is paid to this field.

## Background

Human beings receive most of their knowledge and information through their vision power as one of the vital senses [1]. Eye health is maintained by eyelid function, tear secretion, and prevention of corneal dryness. Tears keep the eye surface moist and destroy microorganisms with antimicrobial components [2]. Hospitalization of patients with an altered level of consciousness in the ICU increases the risk of ocular complications due to the loss of normal protective mechanisms of the eyes, such as decreased tear production and eye blinking reflexes [3]. Patients admitted to ICU require constant supervision and complicated and professional care. Care services are mainly directed at the vital organs such as the respiratory, cardiovascular, and nervous systems [4]. Despite the significance of the ICU in protecting the life of patients [5, 6], ICU patients are exposed to dryness of the eyes, corneal epithelial ulcers, and loss of blinking reflexes, consequently leading to the complete closure of the eye [2, 7, 8]. These complications can cause the spread of infection, corneal perforation, and even loss of vision [1]. Although patients admitted to ICU need care and support for all body systems, most nursing care services are focused on their life-threatening problems, which can reduce the attention and focus of the healthcare provider team on other organs, including the eyes [9]. In addition, most ICU patients need nursing care to maintain their eyes’ natural and pathophysiological health [10]. The results of a study in two teaching hospitals in Iran showed that on the fifth day of hospitalization, 32.2% of the ICU patients had eye dryness and 13.8% had developed corneal wear. Such findings indicate that eye care is ignored for ICU patients [11]. Ebadi (2021) warned about complications caused by lack of eye care in the ICU and reported the prevalence rate of ocular disorders, such as keratopathy, microbial keratitis, and comatose was 3–80%, dry eye syndrome at 33.3%, and eye infections 89.3% [12].

Ocular complications occur in a large number of patients due to the healthcare team’s inattention, neglect, and ineffective care [1, 3, 11]. According to the literature, eye care was ignored by the healthcare team in 62% of ICU patients [7, 13]. However, if eye care is performed regularly, the incidence of superficial eye complications decreases to 8%, confirming the importance of eye care in the ICU [9]. Given the role and importance of eye care in improving the clinical outcomes of ophthalmic patients in the ICU, discovering nurses’ challenges and needs can contribute to finding solutions to patients’ problems, increasing the quality of eye care, and consequently improving the quality of life after discharge of the patients. According to the literature review, the number of studies, especially qualitative ones, is limited in the field of eye care all over the world [2, 10]. Meanwhile, shreds of evidence suggest that nursing care is not performed properly from the viewpoints of patients admitted to the ICU. Thus, identifying the hidden aspects of eye care is of great importance based on nurses’ perceptions and experiences.

Since the qualitative approach, such as conventional content analysis, is a comprehensive, complete, and in-depth way to obtain a clear and accurate picture of a given phenomenon [14], the present study aimed to explore the challenges of Iranian nurses in taking care of ICU patients’ eye health.

## Methods

### Context

#### Nursing staff in Iran

Nursing staff in hospitals in Iran include nurse managers, clinical supervisors, educational supervisors, head nurses, and clinical nurses with an MSc or BSc degree. Operating room nurses and nurse anesthetists with a BSc degree also work in operating rooms. Unlike some western countries, licensed nurses are not ranked in Iran and the registered nurse (RN) is the single professionally accepted rank. The Iranian nursing education program lasts for 4 years and graduates receive a bachelor’s degree. Graduates of nursing schools are considered RN, which is the minimum requirement for nursing practice. Besides, graduated nurses have to work in governmental hospitals for 24 months, after which they are permitted to apply for employment. Enrolment in a master of science program in nursing requires the applicant to hold a bachelor’s degree in nursing and to successfully pass the university entrance exam. The master of science in nursing is a 2–3-year program including about 32 compulsory and optional credits in total [15].

Two other types of health staff are also employed to assist RNs [16]. Nursing is a hard job in Iran because of the high number of patients and the shortage of nurses. The feminine image of a “nurse” is a potential barrier for men considering nursing as a career and the majority of male nurses experienced significant personal, family, and community resistance to entering a nursing profession [17]. Currently, most of the nurses in Iran are females, hold a BSN degree, and work in hospitals and healthcare centers [18]. In 2006, there were 3720 ICU beds in Iran with more than a 90% occupancy rate [19].

Haghdoost et al. (2018) reported that the number of active beds in the country was 117,580 in 2016. They also estimated that to meet the community’s need for healthcare services, this number should reach 194,471 beds by 2026. There were 1.47 beds for 1,000 people in 2016, which will increase to 2.9 in 2026 [20].

### Study design and setting

This study employed a conventional content analysis approach to examine the problems faced by nurses in taking care of the patient’s eyes in the ICU. The purpose of content analysis is to obtain a brief and comprehensive description of the phenomena [21]. The current study tried to analyze the latent content as well as the observable content.

This study was conducted in three hospitals (Bahonar Hospital, Shafa Hospital, and Afzalipour Hospital) affiliated with the Kerman University of Medical Sciences. Each hospital has five ICUs (i.e., Covid-19, poisoning, internal wards (1 and 2), and neurology department) with a total number of 192 staff [22]. This study was conducted from March 2021 to January 2022.

### Sampling, participants, and data collection

In this study, nurses working at hospitals affiliated with the Kerman University of Medical Sciences were interviewed. The participants were selected using the purposeful sampling method [23]. The researcher selected nurses having rich information about the phenomenon. Maximum variation was considered in sampling and nurses were selected with various occupational and demographic characteristics were selected to provide a wide range of information. Inclusion criteria were nurses with a bachelor’s or a higher degree in nursing, being engaged in caring for ICU patients, ability to communicate verbally in Persian, and having at least one year of working experience in ICUs. The exclusion criterion was withdrawal from the study for any reason. Eleven nurses and three head nurses participated. The demographic characteristics of the participants are given in Table 1. The data were collected through interviews and field notes. The researcher first visited the participants and explained the research objectives. The data were collected through open-ended, in-depth semi-structured face-to-face interviews. Open-ended questions in the interview guide allowed the respondents to explain their own experiences and follow-up questions were asked based on the participant’s responses. The majority of interviews were held in the nurses’ restroom in the ICU but two cases were interviewed in the nursing office of the hospital. The interviews were conducted by the first author while all authors reviewed the interviews’ transcriptions and coding processes. After each interview, the researchers reviewed the interviews meticulously and designed more information-seeking questions to be asked in the next interview. The interview continued until the data saturation point where no new codes and categories were obtained. A total of 16 interviews were conducted with 14 participants; two participants were re-interviewed to provide the researcher with the opportunity to develop, confirm, and add new information. The interview questions were about eye care and its challenges. Initially, the interview started with an open-ended question “Can you describe one of your work shifts for me?” Later, the follow-up questions were asked based on the provided information by the participants to clarify the intended concept. At the end of each interview, which lasted about 45 min on average, the interviewee was asked to provide any additional and new information regarding the topic. In the end, a subsequent interview was scheduled after appreciating the participants for their cooperation. All participants attended the interviews and were willing to share their experiences. Table 2 shows examples of the questions that reflected the participants’ experiences of eye care for ICU patients.

**Table 1:** Characteristics ‘participants (*N* = 14)

**Number**	**sex**	**Age**	**ICU Work experiences (year)**	**Level of education**	**Position**	**Type of ICU**	**Participation in the eye care training course**
1	female	29	2	Bachelor’s degree	nurse	general	no
2	female	30	3	Master’s degree	Head nurse	neurology	no
3	female	30	4	Bachelor’s degree	nurse	covid-19	no
4	female	32	5	Bachelor’s degree	nurse	general	no
5	female	35	6	Bachelor’s degree	nurse	poisoning	yes
6	female	36	8	Bachelor’s degree	nurse	covid-19	no
7	female	37	11	Bachelor’s degree	nurse	neurology	no
8	female	38	13	Master’s degree	nurse	general	no
9	female	40	17	Bachelor’s degree	nurse	neurology	no
10	male	41	18	Bachelor’s degree	nurse	covid-19	no
11	female	49	20	Bachelor’s degree	Head nurse	poisoning	no
12	female	50	27	Bachelor’s degree	Head nurse	general	no
13	female	35	6	Bachelor’s degree	nurse	poisoning	no
14	male	38	4	Bachelor’s degree	nurse	general	no

**Table 2:** Examples of questions

1. Can you describe one of your work shifts for me?
2. Is eye care for patients done in your department?
3. How did you respond to the eye care needs of the patients?
4. What conditions did you require to respond better to these needs?
5. What circumstances prevented you from responding to the patient’s eye care needs?
6. Is the eye care you do record in the nursing report? How?

### Data analysis

The current study used MAXQDA-20 to manage data, as well as Graneheim and Lundman’s conventional content analysis method to guide analysis [24]. Thus, themes, categories, and subcategories were extracted from the data. The interviews were digitally recorded, transcribed verbatim, reviewed several times, coded, and analyzed immediately after each interview. Data analysis was performed concurrently and continuously along with data collection. In the present study, each interview served as a unit of analysis. The researcher divided the text into meaning units, each with content and context-related words, phrases, or paragraphs. Table 3 presents examples of the interview content.

**Table 3:** Example of the qualitative content analysis process

**Meaning Unit**	**Condensation**	**Code**	**Subcategories**	**Categories**	**Theme**
“I have been officially hired for 20 years, during this time, we have never received educational information about eye care. This topic has never been raised in educational priorities (P7).	The nurse noticed that is aware of the importance of eye care training for the ICU personnel	Inadequate training for nurses -Insufficient attention of the head nurses to the training needs of personnel	Nurse’s low cognitive skills	Inadequate clinical and professional competence of nurses	Instances of lack of evidence-based approach
“Most nurses do not know about the exact part of the eye that should receive medication, the interval between medications, and the symptoms that should be reported to the physician. Although training these information and eye infection prevention methods to nurses is important, usually, more vital training is considered in educational needs assessment and eye care has been neglected.“ (P4)	Head nurses admit to inadequate knowledge of nurses in the field of eye care and how to provide standard and principled care	-Inappropriate educational needs assessment			

To perform initial coding, the participants’ statements were examined carefully by the researchers to extract meaning units and concepts from the transcriptions in the form of initial codes. These codes were reviewed several times and those with similar contents were merged into more solid sub-categories reflecting the nurses’ experiences and challenges in caring for the patient’s eyes. Next, separate subcategories were studied and merged into broader categories, which were merged to form the final theme. The codes and meaning units were examined and revised from data analysis up to the final stages of the research project by all authors to ensure the accuracy of the findings. Although the analysis process was systematic, a back-and-forth movement was available between the whole and the components of the text. Table 4 summarizes all categories and subcategories extracted in this study.

**Table 4:** Themes, categories, subcategories and codes extracted from qualitative content analysis

**Codes**	**Subcategories**	**Categories**	**Themes**
Inadequate training for nurses Improper educational needs assessment Lack of understanding of the importance of eye care in university education Inadequate knowledge of the nurse	The lack of education importance	education, the missing loop	Effects of lack of evidence-based care approach
Nurses’ misconceptions Employing a novice nurse in the ICU The role of initial assessment in determining the need for eye care Inadequate communication skills Low self-confidence of the nurse	Incompetence of the nurse in performing assigned duties	Inadequate clinical competence of the nurse	
Failure to pay attention to the consequences of improper eye care Handing over patient’s eye care to paramedics Providing inadequate care Lack of attention to preventive care Check only pupil reaction Not requesting ophthalmology consultation to implement preventive exercise The attitude of taking action after the appearance of eye symptoms	Negligence in eye care by the nurse		
Lack of eye care protocol Lack of clinical guidelines for eye care Lack of educational pamphlets Care according to taste Experience-based care	Care based on tradition and experience	Unsafe nursing care	
Different performance of nurses in eye care of patients with suction Different implementation of nursing interventions in patients with high oxygen flow Incomplete implementation of eye care based on the nursing process Using wrong nursing diagnoses in the patient’s initial assessment sheet	Inconsistency of nursing care with scientific principles		
Defects in recording eye health in the initial assessment sheet Defects in the transfer sheet from ward to ward Defects in the nursing report Failure to register in Cardex	Low importance of eye care in patient file documentation	Difficult evaluation of eye care	
Examining eye health status based on experience Estimating eye damage based on nurses’ mentalities Lack of visual tools in estimating the degree of eye damage Failure to work with an ophthalmoscope	Subjectivity of eye health assessment		
Inadequate attention of the clinical supervisor to the way of eye care by the personnel The presence of eye medicines in the form of ointments and drops Tick is the only way to record eye care measures Not including the eye consequences in the Accreditation criteria	Not including eye care in management evaluations		
The nurse waiting for the doctor’s attention and attention to the eyes (this is not my job) Depending on the doctor’s order The nurse’s interest in receiving instructions on eye care from the doctor Look up and down Passive behavior of the nurse	Doctor-oriented	Organizational requirements	
Reduction of motivation due to lack of encouragement from nurses lack of appreciation from superiors to nurses, A flaw in the reward system	Inadequate support from managers		
critically ill Overcrowding of the intensive care unit Lack of manpower	Inappropriate working conditions		

### Trustworthiness of data

In 1994, Guba and Lincoln determined credibility, dependability, transferability, and confirmability as practical techniques to gain the robustness of data [25]. Research robustness was established through the researcher’s deep involvement in the discovery and confirmation of the data [26] to reflect the participants’ experiences accurately. To promote the credibility and accuracy of the findings, several methods including in-depth interviews, member checks or respondent validation, and content analysis were triangulated. To ensure the findings’ dependability and stability, the recorded files were transcribed word-by-word immediately after each interview and the transcriptions underwent peer review and member check. Furthermore, the accuracy of the interpretations and coding process was corroborated by recording the data collection and code extraction processes verbatim. To ensure confirmability, all details, including the data collection and analysis procedures were carefully recorded. The participants were selected with maximum variation in terms of demographic and occupational characteristics. Two experts in qualitative research checked the research process and findings. Moreover, the research documents including the raw data, notes, transcripts, and recorded audio of the interviews were stored for possible future reviews. For the transferability of data, details regarding the methodology were described accurately to use findings in other contexts and for future considerations.

## Results

The participants’ mean age was 37.14 ± 6.41 years and their average work experience in ICU was 10.29 ± 7.63 years. Most participants were women (85.7%) and 14.3% had postgraduate education.

From a total of 16 interviews, 410 primary codes were found. Given the semantic approximation, many codes were merged. Finally, the “lack of an evidence-based approach” was identified as the core theme. It covered five categories including “education, the missing link”, “nurses’ inadequate professional competence”, “unsafe nursing care”, “organizational requirements”, and “difficulty in eye care evaluation”.

### The core category: The lack of an evidence-based approach

#### 1. Education, the missing link

According to the nurses, inadequate eye care was among the main challenges in taking care of the ICU patients’ eyes. They believed that although education is required to empower nurses in eye care, nursing managers do not care about its significance due to their lack of awareness about implementing eye care. Other reasons for inadequate attention to nurses’ education were “the lower importance of eye care in the patient’s life-threatening conditions, ineffective needs assessment of ICU nurses’ educational needs, and authorities’ unawareness of educational methods or educational content”. According to the findings, nurses need to obtain information about assessing the patient’s eye health, the frequency, and site of using eye ointments and drops, and perform preventive care for ocular complications caused by patients’ admission to the ICU. However, the nurses stated that they did not receive any education in the field of eye care through the formal educational system. They also complained that eye care was not emphasized by the relevant managers and authorities in their working units.

Accordingly, a participant mentioned, “*I have been officially hired for 20 years. We never had a training course about eye care. This topic was not addressed in any educational programs*” (P7).

Another nurse stated, “*Most nurses do not know about the exact site of the eye that should receive medication, the interval between medications, and the symptoms that should be reported to the physician. Although providing these instructions and eye infection prevention methods to nurses is important, usually more vital training should be incorporated into educational needs assessment, and eye care has been neglected”* (P4).

Despite the existence of several theoretical and practical courses on intensive care in nursing education programs, unfortunately, eye care education has been marginalized and downplayed in academic nursing education. In other words, more attention was paid to the management of the patient’s life-threatening conditions: “*When we were students, we never received any education about eye care. Most educational contents were about suctioning, oxygen levels, and the Glasgow Coma Scale (GCS). We did not receive any training about eye care in the college” (P3)*.

#### 2. Nurses’ inadequate professional competence

Analysis of the participants’ experiences showed that nurses did not have adequate professional competencies in eye care. Professional competency is defined as a set of characteristics in nurses that improve the quality of nursing care and care outcomes. Some reported some competency-related problems such as inadequate knowledge, inefficiency in performing duties, negligence in eye care, inadequate communication skills, low self-esteem, and lack of teamwork.

A participant with 27 years of work experience in the ICU said, “*A nurse must have a professional conscience and clinical reasoning skills to provide effective care to patients*” (P5).

Another participant with eight years of experience in the ICU stated, “*If a nurse has enough knowledge of eye care, she can effectively assess the patient’s needs and conduct principled care because the initial assessment determines the routine care performed for the eye”* (P8).

According to the participants’ statements, nurses underestimated their knowledge, skills, and abilities and believed that eye checks should be performed by ophthalmologists. As they stated, nurses should only perform pupil checks. This confirms their low self-esteem, inadequate interdisciplinary communication skills, and passive performance in patient care. In addition, nurses considered eye care, the use of drops or ointments, and closing the patient’s eyelids as duties of nurse assistants, not nurses. The participants commonly stated, “*We only control the patients’ pupils by flashlight. More thorough examinations should be performed by physicians and they would obtain ophthalmologists’ advice if necessary. Generally speaking, assistant nurses are responsible for using drops and ointments for the patient’s eye or closing the patient’s eyes with adhesive tapes”* (P5)

In the same vein, another participant stated, “*Unfortunately, the nurse-to-nurse and nurse-to-physician relationships are not good. That is to say, nurses are ashamed to ask more experienced nurses or doctors about the patient’s eye care. For example, they are afraid to seek help and advice for the patient’s problems. In such cases, the nurse leaves the patient believing that in the next shift, others will do something for him/her. We have seen such cases*” (P1)

#### 3. Unsafe nursing care

The participants’ experiences indicated that the lack of an evidence-based approach leads to unsafe care. Nurses stated that patient eye care in the ICU is performed based on the personnel’s ideas and experiences, nursing interventions not consistent with scientific principles, as well as the different procedures in eye care. A participant with 10 years of experience stated, “*Eye care in our ward is performed by a normal saline wash. For example, every morning before the shifts change, we clean the patient’s eyes with normal saline and sterile gas. Only in the case of patients with low GCS, we close the patient’s eye with anti-allergic adhesive tapes*” (P1).

A nurse with 17 years of job experience in the ICU indicated, “*For eye care, we don’t usually pay too much attention to the patient’s eyes at the time of admission to the hospital. We had a patient with completely swollen conjunctivitis. I knew from experience that these patients usually get worse and die. To take care of these patients’ eyes, we just tape their eyes closed”* (P2).

Based on the nurses’ experiences, factors, such as high workload, the shortage of nurses, and crowded wards result in unsafe and unprincipled care in ICU. A male participant with two years of experience in the ICU said “*Since nurses are faced with a huge workload in the ICU, they cannot do pupil checks every two hours. So, we only check the patients’ pupils twice a day; at the beginning and the end of each shift*” (P11).

#### 4. Organizational requirements

Many participants were more attentive to the scope of their occupational duties in addition to performing the routine care plans and physician orders. Although some nurses acknowledged their abilities to go beyond their job descriptions, they only made decisions within the scope of their duties because they would be questioned by doctors and prosecuted by the law if they did something not included in their job descriptions.

The nurses stated that they examined the patient, determined the problem, and reacted to it if the treatment was part of their job description; otherwise, they would refuse to take any treatment measures and inform the doctor.

One of the head nurses of the ICU suggested, “*Sometimes the patient’s eyes may have developmental secretion and pus despite the application of ointment. In this case, we should inform the doctor and act upon the ophthalmologist’s order. We cannot give a drop of water to the patients without the physician’s permission”* (P4).

*“Simple ophthalmic care is one of the orders that should be performed perfectly. The only thing that matters for us is the doctor’s order*” (P13).

Although the participants emphasized paying attention to the patient’s needs in nursing care, it seems that physicians are mainly considered a reference for diagnosing the client’s needs without taking into account the patient’s values, preferences, and concerns. This indicates the gap between modern and traditional nursing. In other terms, while modern nursing necessitates conducting evidence-based care, performing patient-centered care, paying special attention to the patient’s needs, and involving them in the treatment process, nursing in our national hospital system emphasizes the traditional care and implementation of medical instructions. In this way, the doctor’s instructions and routine tasks establish the basis of care.

#### 5. Difficulty in eye care evaluation

Evaluation of nurses’ performance in terms of eye care is faced with challenges by officials and head nurses. Head nurses stated three main reasons for difficulties in evaluating eye care: (1) Instead of writing a nursing report, nurses tick on the patient’s sheet and consider it a suitable alternative to documentation of eye care in the ICU. Consequently, they do not mention the eye care measures taken in their nursing report. (2) Given that ophthalmic drugs are often used in the form of drops and ointments, assessing their quantity and quality is hard for the supervisors because it is difficult to measure and estimate the volume of drugs used or unused by nurses for ICU patients. (3) Since patients admitted to ICU are not often able to report the quality of nursing care, especially their eye care, evaluation of nursing interventions in the field of eye care has been accompanied by problems. A participant referred to the quality of nursing care for the ICU patients’ eyes, “*When writing a nursing report for eye care, we just tick the options. If we see a particular point and if special treatment is required, we write a note like ‘eye drops are shed, the eye doesn’t have an infectious discharge, etc.’ Otherwise, as usual, we just tick the options and that’s all” (P10)*.

*“The nurse would often come, pick up the ICU sheet, tick on it, and leave the shift. That is to say, there will be no follow-up procedure, evaluation, and care for the patient. So, it’s really very difficult for me as a head nurse to understand why the patient, who received eye care based on the documentation, hasn’t recovered yet*”. (P14)

Participants’ statements regarding eye care assessment indicated concerns about the adequacy of nurses’ attention to the patient’s eye in the initial evaluation in the ICU or deficiencies in the quality of ophthalmological nursing reports. Excerpts from the participants’ statements are as follows:

“*When the patient is hospitalized, we can do our initial assessment up to the end of our working shift. The initial evaluation involves assessing the patient’s physical and mental status, factors threatening their safety status, etc. But everything depends on whether the evaluating nurse cares if the patient’s eye is healthy. Sometimes, the patient has been in the ward for two days and no one has noticed the patient’s eye problem; everything (from initial care to treatment) depends on the knowledge and information of that nurse”* (P4).

The participants also acknowledged that the initial assessment and consequently the eye care procedures in the ICU were not desirable. They urged further consideration and training in this area.

A nurse stated, “*We need some education about the importance of using the nursing process in eye care and most importantly, the significance of the patient’s initial assessment by nurses during patient care*”. (P12)

Thus, the participants’ statements confirmed the significance of ‘patient’s eye care’ amid the complex and numerous problems of the patient and highlighted the necessity of employing holistic and multidimensional nursing care for ICU patients.

## Discussion

An exploration of the nurses’ experiences highlighted the absence of an evidence-based approach” to ICU patients’ eye care. The findings revealed the importance of nurses’ experiences and the challenges faced by them in taking care of the patient’s eyes.

Unfortunately, eye care has been neglected not only in Iran but also around the world. The ICU nurses believed that ICU staff mostly take care of the patients’ life-threatening signs and symptoms [27]. This viewpoint has paved the way for nurses to let evidence-based care, as one of the main components of health services, fall into the abyss of oblivion.

Since the ultimate goal of nursing services is to provide quality care to improve the outcome of services to the patients and the community, many medical and care measures are taken based solely on traditional trends, guesses, occupational assumptions, individual skills, and non-organized clinical observations. As a result, health system services are expected to be based on evidence, scientific methods, and scientific decision-making [28]. Evidence-based practice is defined as performing the best and most correct care in the best and most efficient way and at the best time for the patient. It also emphasizes the conscious, explicit, and judgmental use of the best new shreds of evidence to make decisions about caring for each patient [29]. This type of practice is essential for nurses and the nursing profession because it helps nurses build their own body of knowledge, minimize the gap between nursing education/research and practice, and standardize their nursing care [30, 31]. On the one hand, nurses’ clinical decisions should be made based on the best and most up-to-date research evidence available [32]. Patients reasonably expect healthcare services to be based on the most up-to-date scientific findings [33].

The results of this study indicated that nursing care programs for eye care are not developed and implemented based on scientific evidence in Iran because nursing eye care starts after the eye damage has occurred and only medications prescribed by the physicians should be applied to the patient. According to Momeni Mehrjardi (2021), in some countries, nursing care for the ICU patients’ eyes is not based on an evidence-based approach”[9]. In Iran, nurses have low-to-moderate levels of knowledge about evidence-based practice and do not often use evidence-based practice in patient care [34]. The frequency of evidence-based practice was reported to be 42% [35] in Oman and 14% in Saudi Arabia [36]. Stillwell (2010) also stated that many nurses are not able to implement evidence-based nursing care [37]. According to Mehrdad et al. (as cited in Pashaypour, 2016), 85.9% of nurses are poorly prepared to use scientific evidence and research findings in their clinical practice [33].

In this study, we found that “education is a missing link” and one of the challenges faced by nurses in taking care of patients’ eyes, highlighting the absence of an evidence-based approach.

The nurses’ did not possess adequate knowledge of eye care, which can be associated with inadequate nursing education in the field of eye care, ineffective assessment of nurses’ educational needs, unawareness of the importance of eye care for ICU patients in academic education, and the failure to pay attention of the necessity of implementing holistic and humanitarian care in nursing faculties, as indicated in the literature [2, 12, 38, 39]. However, some studies reported that nurses had good knowledge of eye care [40, 41]. Discrepancies in these findings could be attributed to the differences in education, work experience, and skills of the participants and the employed research design. Furthermore, some studies employed a quantitative approach and examined the ICU nurses’ knowledge through questionnaires. Thus, we cannot argue that the knowledge obtained from quantitative surveys is a good measure of nursing performance or the quality of nursing care. These findings have been derived from different sources and, thus, more qualitative and quantitative research is needed to establish a relationship between these variables [41]. Therefore, eye care training should be incorporated into intensive care training programs for nurses to help them enhance the quality of patient care.

The “nurses’ inadequate professional competence” was associated with nurses’ inadequate knowledge, nurses’ inefficiency in performing the assigned duties, and nurses’ negligence in eye care. The holism theory considers competency as a set of factors, including knowledge, skills, attitudes, thinking ability, and values needed in specific fields [42]. Krokmyrdal (2015) defines clinical competency as the essence of the nursing profession and the foundation of effective and superior performance required in a profession or a clinical position [43]. Qualitative studies in this field have reported internal or individual, and environmental factors as the main factors affecting the clinical competency process [44]. Consequently, authorities should keep in mind that environmental and organizational factors including educational facilities, in-service retraining, and training programs, as well as an efficient system of control, supervision, and education can affect nurses’ clinical competency.

One of the challenges reported by the nurses in this study was unsafe nursing care. Accordingly, scholars believe that the first requirement for safe care in ICU is to meet the standards, and if these requirements are not met, optimal and quality care cannot be provided, resulting in unsafe care [45]. Another study in Iran listed the effective factors for unsafe care, such as nurses’ inadequate knowledge about safe drug delivery, high work pressure, incorrect safety culture in different wards, and inappropriate policy-making in ensuring patient safety [46]. In other terms, patients are provided with safe care wherever nurses act professionally, while unsafe care occurs when the nurses do not act professionally. In the present study, care provision based on nurses’ ideas and experiences as well as non-conformity of nursing care with scientific principles was one of the characteristics of non-professional nurses’ performance followed by errors and unsafe care.

Difficulty in evaluating eye care was another challenge reported by the participants. According to Asgari, “the first and most important factor in improving the quality of care is the evaluation of nurses’ performance in providing nursing care to patients”[47]. Evaluation of nurses’ activities is of great importance as it contributes to specifying the areas that need to be upgraded, identifying nurses’ educational needs, and ensuring the optimal provision of care. Such assessment is referred to as the gravity point of performance of quality assurance systems, workforce planning, and human resource management [48]. In this vein, the evaluation of nurses’ viewpoints can represent different dimensions of variables affecting professional indicators and the quality of nursing care, which eventually facilitates improving the quality of nursing care [47].

According to the nurses’ experiences, “organizational requirements” were associated with physician-centeredness, inadequate support from managers, and unfavorable working conditions that were caused by the absence of an evidence-based approach. Organizational requirements play a clear and significant role in the care process. Other studies indicated that organizational requirements are of great importance in providing quality care. According to Bucknall, although nurses consult doctors in cases where they cannot make decisions legally and independently, it is the doctors who make the final decisions [49].

Nursing is one of the challenging jobs because nurses have daily interactions with patients, colleagues, and other health professionals but most nurses do not have many opportunities to express their opinions since they have to render care services for long hours in a tense atmosphere and enforce strict regulations. Nursing, as a critical, ambiguous, and transient occupation, has largely been downgraded in comparison with other medical professions [50].

Coombs (2003) argues that the key structure and content of relationships between physicians and nurses to make decisions in intensive care are strongly influenced by basic knowledge and medical authority [51]. It seems that paying attention to nurses’ workload and enabling the active participation of nurses in clinical decision-making will ensure the improvement of quality of care and optimal performance in ICUs.

### Limitations

One of the limitations of this study was that we only focused on nurses’ perspectives; however, the views of ICU doctors or the patient’s family could also provide richer information. Therefore, future studies can explore ICU physicians’ or family patients’ perspectives on eye care. As another limitation, this study was conducted on ICU nurses in hospitals affiliated with KUMS. It is recommended to conduct similar studies on nurses working in private hospitals and other wards and compare the results with the findings of the present study.

## Conclusion

Following the findings of this study, caring for ICU patients’ eyes has been neglected due to the lack of evidence-based practice. Thus, the Ministry of Health and Medical Education of Iran is required to prepare localized clinical guidelines and make appropriate fundamental decisions. If necessary, a directive can be issued to require hospitals to engage healthcare providers, especially nurses in the decision-making process. A clear guideline can improve the quality of nurses’ eye care and subsequently increase patient safety. Moreover, nursing education planners and educational managers are needed to revise the nursing education programs and academic syllabi to provide the necessary ground for the education of principled and correct eye care in academic and in-service training programs. Nursing managers can enhance evidence-based practice in clinical settings by creating a suitable organizational environment, adopting practical policies, enacting laws and regulations to facilitate evidence-based practice, and improving nurses’ competencies by creating a suitable platform for evidence-based practice.

## Data Availability

The datasets used and/or analyzed during the current study are available from the corresponding author upon reasonable request.

## Abbreviations

EBP: Evidence-Based Practice
GCS: Glasgow Coma Scale
ICU: Intensive Care Unite
